# Ketamine-induced uropathy: A diagnostic pitfall in an increasing healthcare issue in youngsters

**DOI:** 10.1016/j.eucr.2022.102019

**Published:** 2022-02-01

**Authors:** Glenn Lamers, Johan Van Dyck, Stijn Schapmans, Katrien De Coster, Dries Mortier, Natalia Zabegalina

**Affiliations:** a Department of Urology, Maria Hospital North Limburg, Maesensveld 1, 3900, Pelt, Belgium; b Department of Urology, University Hospitals Leuven, Herestraat 49, 3000, Leuven, Belgium; c Department of Urology, AZ Vesalius, Hazelereik 51, 3700, Tongeren, Belgium

**Keywords:** Bladder, Ketamine, LUTS, Uropathy, ADHD, Attention-deficit/hyperactivity disorder, BPS, bladder pain syndrome, CRP, *C*-reactive protein, hpf, high power field, IV, intravenous, KIU, ketamine induced uropathy, LUTS, lower urinary tract symptoms, NMDA, *N*-Methyl-d-aspartate, STD, sexual transmitted disease, UTI, urinary tract infection

## Abstract

Ketamine induced uropathy (KIU) is a urological condition increasing in prevalence, with similar symptoms to UTI, OAB syndrome or interstitial cystitis/bladder pain syndrome. We present the case of an 18-year old male who established severe LUTS and acute kidney injury due to KIU, in a short time-span of 6 months. Since cessation of ketamine is the cornerstone of treating KIU, correct and early diagnosis is essential. Physicians should therefore consider KIU as a differential diagnosis in storage LUTS, especially in younger patients with therapy-resistant LUTS.

## 1 Section headings

• Inflammation and Infection
• Male Lower Urinary Tract Symptoms
• General Urology

## 2 Introduction

Ketamine finds its roots back as an anaesthetic drug acting through NMDA-receptor blockage. Its medical use is however limited because of the delirious side-effects. Even though the medical use is diminished, ketamine found its way into the recreational environment as a party drug. Known as ‘K’, ‘special/vitamin K’, and ‘super acid’, it produces a short-term out-of-body experience also described as ‘the K-hole’.^1^ Ketamine abuse could however lead to ketamine-induced uropathy (KIU). The urinary bladder is affected in most cases and symptoms can resemble conditions such as urinary tract infection (UTI), ulcerative or interstitial cystitis (IC), overactive bladder syndrome and bladder pain syndrome (BPS).^2^ Diagnosing the disease can therefore be a challenge.

## 3 Case report

We present the case of a 18-year old male with a history of ADHD and drug abuse. He started using ketamine in February 2020, consuming around 7 g per week. His abuse severely increased to 20–30 g during the national COVID-19 lockdown.

He presented at the urological outpatient clinic in September 2020, complaining of suprapubic pain, storage LUTS (urgency, daytime frequency, nocturia) and intermittent urinary incontinence for several months. He also complained of sporadic gross haematuria. There were no voiding LUTS.

Urine analysis showed microscopic haematuria (23/hpf) and severe pyuria (1133/hpf). Urine culture and cytology were negative. STD-screening was negative and ultrasound of the urinary tract was normal. He had a very low bladder capacity of 18 cc. Cystoscopy showed an ulcerative cystitis.

Solifenacin 10mg once daily was started and immediate cessation of ketamine was advised.

One month later he presented at the emergency department with bilateral flank pain without fever. Creatinine and CRP levels were severely increased to 1.66 mg/dL and 211 mg/L respectively. Imaging showed bilateral hydroureteronephrosis as well as a shrivelled bladder. He was admitted for IV-antibiotics. Fesoterodine 8mg and mirabegron 50mg were started to maximally decrease bladder overactivity.

**Fig. 1:**
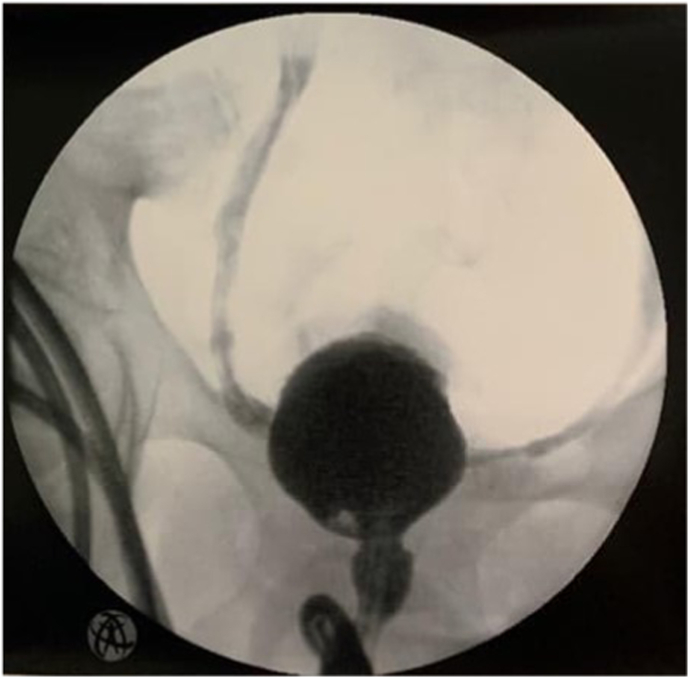
Cystography with 50 cc of contrast. Note the leakage of contrast along the urethra and bilateral reflux indicating the severely decreased bladder capacity.

Further deterioration of kidney function and persistent bilateral hydronephrosis were seen the next day. Cystoscopy under general anaesthesia was performed, which showed a diffuse ulceration of the urothelial mucosa and submucosal bleeding. Cystography revealed bilateral reflux and urethral leakage after injection of 50 cc of contrast, emphasising the severe decrease in bladder compliance (Fig. 1).

**Fig. 2:**
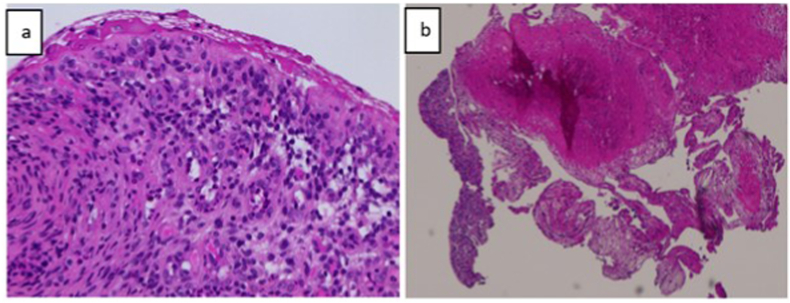
Increased number inflammatory cells with loss or superficial urothelial cell layer is shown in a. In b, marked fibrin deposition is shown indicating the increased activity of submucosal fibroblasts.

Bilateral ureteral stents were placed, bladder biopsies were taken and a Foley catheter was left behind. The bladder biopsy showed chronic inflammation with widespread denudation of the urothelial wall. It also showed thickened blood vessels and fibroblast proliferation in the submucosal layer (Fig. 2).

**Fig. 3:**
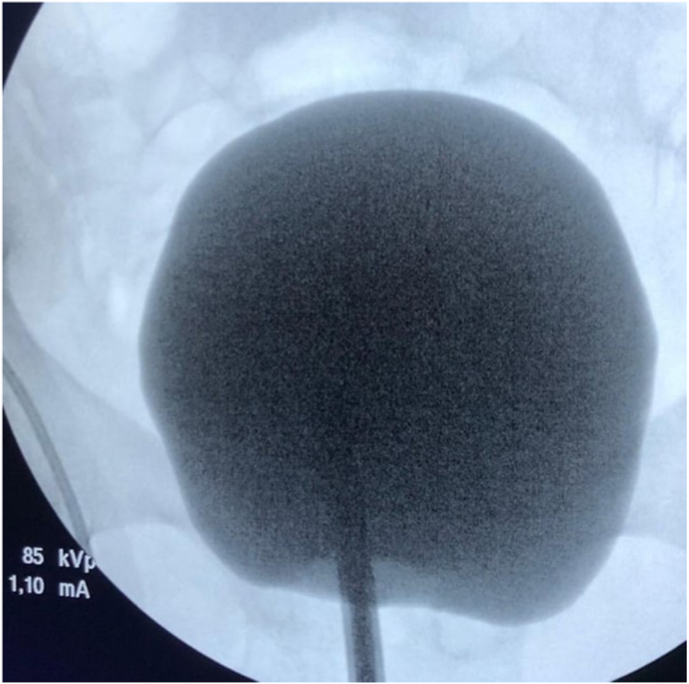
Cystography after several months of abstinence and symptomatic treatment. Bladder capacity up till 300 cc without signs of ureteral reflux or urethral leakage.

Renal function soon improved afterwards. The Foley catheter was switched to a condom catheter several days later to manage the urinary loss. After discharge, his symptoms improved over the following months. The urinary incontinence resolved and the storage LUTS were managed by a single anticholinergic agent. Therefore urodynamic investigations were not performed at re-evaluation. The ureteral stents were removed and a repeat cystography showed a bladder capacity of 300 cc without signs of ureteral reflux or urethral leakage (Fig. 3).

## 4 Discussion

Ketamine-induced uropathy is an urologic condition with an increasing incidence over the last decade. In a recent survey conducted in the UK, ketamine-abuse in people aged 16–24 years has increased from 1.9% in 2008/2009 to 2.9% in 2018/2019.^3^ In Asian countries such as China and Taiwan, up to 40% of drug users use ketamine. In 2014, more than 2000 cases of ketamine abuse were reported in Hong Kong alone.^4^

The clinical presentation of KIU can vary. Roughly 50% experiences LUTS, primarily of the storage type including frequency with small voided volumes, urgency, nocturia and dysuria.^5^ Patients sometimes experience pelvic pain, at rest or during voiding, and urgency incontinence. Gross haematuria is sometimes present as well.

KIU and its symptoms seems to be influenced by the dose, frequency and duration of the abuse. Although discussion exists about the causal relationship between ketamine and the LUTS, it seems unlikely that other substances taken in conjunction with ketamine would cause the symptoms. This is because using different adulterants still establishes the disease with ketamine as the common factor and several case reports of KIU exist of patients using ketamine in different clinical settings.^1^

The mechanism of action is not fully understood but seems to consist of multiple pathways including direct toxicity of metabolites to the mucosa, causing chronic inflammation and fibrosis, microvascular damage and neurogenic inflammation.^1^

Ketamine can mitigate the symptoms temporarily, risking development to more advanced stages of KIU. Furthermore, the clinical presentation can resemble that of more common conditions such as UTI, with positive urine cultures in up to 30% of patients due to secondary infection.^4^ Since antibiotics do not treat the underlying condition, many patients risk delay in diagnosis and management.

Castellani et al. recommend urine analysis and culture together with renal and bladder ultrasound as first-line investigations in patients between 16 and 30 years old presenting with storage LUTS and pelvic pain.^4^ We would propose adding early assessment of potential ketamine abuse at first presentation, especially in patients with a history of substance abuse, instead of waiting until other diagnoses are excluded or when imaging shows abnormalities. When ketamine use is established, cystoscopy should not be systematically performed in these patients, except when vesical abnormalities on ultrasound or gross haematuria are present, like in this case. If cystoscopy is abnormal, urine cytology can be performed to exclude carcinoma in situ. Since KIU is still relatively rare, it should not be at the top of the list of differential diagnoses when a younger patient complains of storage LUTS. Physicians should however be aware of the possibility, especially when symptoms won't improve.

The cornerstone of treating KIU is cessation of ketamine and management of its symptoms. Most patients respond to anti-inflammatory, anticholinergic and analgesic agents. Second line treatment consists of intravesical instillations, bladder hydrodistension or Botox-injections. In severe cases, (partial) cystectomy and reconstruction is sometimes necessary to alleviate symptoms.

## 5 Conclusion

Ketamine-induced uropathy is an increasing health care issue warranting early and correct diagnosis for proper management. Its clinical presentation can however resemble common urologic pathologies and KIU should be considered in the differential diagnosis to prevent diagnostic delay.

## 6 Informed consent

Provided by the patient.

## Funding source

This research did not receive any specific grant from funding agencies in the public, commercial, or not-for-profit sectors.

## Author contributions

Glenn Lamers: Conceptualization, Writing - original draft. Natalia Zabegalina: Writing - review & editing, Supervision. Natalia Zabegalina, Johan Van Dyck, Stijn Schapmans, Katrien De Coster, Dries Mortier: Writing - review & editing.

## Declaration of competing interest

The authors declare that they have no financial conflict of interest with regard to the content of this report.
